# RETRACTION: Plasma Small Extracellular Vesicle‐Carried miRNA‐501‐5p Promotes Vascular Smooth Muscle Cell Phenotypic Modulation‐Mediated In‐Stent Restenosis

**DOI:** 10.1155/omcl/9806752

**Published:** 2026-06-23

**Authors:** Oxidative Medicine and Cellular Longevity

RETRACTION: X. F. Gao, Z. M. Wang, A. Q. Chen, et al., Plasma Small Extracellular Vesicle‐Carried miRNA‐501‐5p Promotes Vascular Smooth Muscle Cell Phenotypic Modulation‐Mediated In‐Stent Restenosis, Oxidative Medicine and Cellular Longevity, 2021, 6644970, 20 pages, 2021 https://doi.org/10.1155/2021/6644970.

The above article, published online on 22nd April 2021 in Wiley Online Library (wileyonlinelibrary.com), has been retracted by agreement between Editor in Chief Jeannette Vasquez‐Vivar; and John Wiley & Sons Ltd. The retraction has been agreed due to figure concerns initially raised on PubPeer [1]. Specifically:

• Image duplication was detected in FIGURE 4e: “control” and “mimic‐NC” are different experimental groups and have received different treatments. The Transwell assay images from these two groups were unexpectedly similar.
• Image duplication was also detected in FIGURE 4j, “ISR‐sEV” group vs. “ISR‐sEV+ inhibitor‐NC” group.

Through an investigation, the publisher confirmed these two concerns and observed additional image inconsistencies:

• Image duplication was detected in FIGURE 4i, where two sections of the fluorescence image, “ISR‐sEV” (middle panel) and “ISR‐sEV + inhibitor‐NC” (right panel), appear unexpectedly similar but are under different experimental conditions.

The authors were unable to provide the original data images in a condition that the publisher and editor could evaluate and verify. The explanations given by the authors were considered insufficient. Therefore, the results are considered unreliable and the article is retracted.

The authors disagree with the retraction.
